# RPDL-YOLO: An efficient YOLOv8 framework for small bird detection in airport airside environments

**DOI:** 10.1371/journal.pone.0362491

**Published:** 2026-11-12

**Authors:** Xinling Wen, Liming Liu, Yu Chen, Zhaoyu Liu, Zhengxiang Ma, Yapeng Wu, Kun Liang, Teng Jiang, Heng Yang

**Affiliations:** 1 School of Electronic and Information, Zhengzhou University of Aeronautics, Zhengzhou, Henan, China; 2 Henan Key Laboratory of General Aviation Technology, Zhengzhou University of Aeronautics, Zhengzhou, Henan, China; 3 Collaborative Innovation Center of Aeronautics and Astronautics Electronic Information Technology, Zhengzhou, Henan, China; 4 The Airfield Area Management Department, Xinzheng International Airport, Zhengzhou, Henan, China; MVSR Engineering College, INDIA

## Abstract

Bird intrusions into airport security zones pose significant threats to aviation safety. However, the small size, rapid movement, and irregular flight patterns of birds often result in missed detections and false alarms in vision-based surveillance systems, especially in complex open-sky environments with low contrast and significant background interference. Although YOLOv8s achieves strong real-time performance, it remains limited in fine-grained feature representation and small-object detection capability. To address these challenges, we propose an improved detection model termed **RPDL-YOLO** (Refined Perception with Dual-branch Detection and Localization YOLO). The proposed model introduces three key enhancements: (1) integrating RepViTBlock into the backbone to improve spatial feature representation; (2) removing the redundant P5 detection head and constructing a dual-branch high-resolution P2 detection structure to strengthen small-object perception; and (3) designing a Mix_IoU loss function that combines EIoU and GIoU to improve localization accuracy and training stability. Experimental results demonstrate that, compared with the YOLOv8s baseline, the proposed method achieves improvements of 7.6% in Precision, 5.4% in Recall, and 4.6% in mAP@50, while maintaining a real-time inference speed of 93.1 FPS. These results demonstrate that RPDL-YOLO significantly enhances detection performance for small and fast-moving bird targets while maintaining computational efficiency, making it suitable for practical airport bird-strike prevention.

## Introduction

Bird strikes constitute a long-standing and critical threat to global aviation safety. According to industry statistics, bird strike incidents result in billions of dollars in annual economic losses due to aircraft damage, flight delays, and maintenance costs, and in rare but severe cases, may even contribute to catastrophic aviation accidents [1,2]. With the continuous growth of air traffic and the expansion of airport infrastructure into surrounding wildlife habitats, both the frequency and potential severity of such events have shown an increasing trend, particularly during takeoff and landing phases when aircraft are most vulnerable.

With the rapid development of deep learning, vision-based detection methods have achieved significant progress, among whice, the YOLO (You Only Look Once) series has become one of the most widely adopted frameworks due to its favorable balance between detection accuracy and computational efficiency. Recent studies have applied YOLO-based models to bird detection in UAV and airport scenarios [3–5]. However, reliable bird detection in real-world airport environments remains challenging. Aggressive downsampling in YOLO-based models leads to the loss of fine-grained spatial details, which are essential for distinguishing tiny birds from visually similar background patterns. In addition, high-resolution feature representations (e.g., P2) are often underutilized, resulting in reduced sensitivity to sub-10-pixel targets. Furthermore, bounding box regression for extremely small objects is inherently unstable, as low overlap between predictions and ground truth produces weak and noisy optimization signals. These challenges are further exacerbated by the limited availability of large-scale and diverse airport bird datasets, resulting in limited generalization across different environmental conditions. These limitations jointly hinder the reliable detection of small and distant bird targets in real-world airport environments.

To bridge this gap, we propose the RPDL-YOLO framework, a task-oriented detection model specifically designed for airport bird monitoring. The proposed framework integrates three complementary innovations, each targeting a critical bottleneck in existing YOLOv8-based detection pipelines:

- We propose a backbone context enhancement mechanism by selectively integrating RepViTBlock into the C2f modules of YOLOv8. This hybrid design introduces lightweight transformer-style feature mixing into convolutional pathways, enhancing fine-grained spatial modeling without altering the original network topology or computational budget.
- We introduce a dual-path detection architecture at the native P2 resolution to address the severe feature degradation of small birds caused by aggressive downsampling. By decoupling high-resolution feature streams, the proposed design strengthens the representation of minute objects while maintaining compatibility with multi-scale detection.
- We develop a hybrid IoU regression loss function, termed Mix_IoU, by combining EIoU and GIoU with tunable weighting. This formulation jointly considers center distance, aspect ratio, scale consistency, and enclosing geometry, providing a more balanced optimization objective for bounding box regression.

The remainder of this paper is organized as follows. Section 2 reviews related work on bird detection and small-object detection in airport environments. Section 3 presents the proposed RPDL-YOLO model, including the backbone improvement, dual-branch detection head, and loss function design. Section 4 describes the experimental setup and reports ablation studies as well as comparative results. Finally, Section 5 concludes the paper and outlines future research directions.

## Related work

Recent advances in deep learning have spurred increasing interest in vision-based bird detection for aviation safety. Existing studies can be broadly categorized into several research directions, including dataset construction, YOLO-based architectural improvements, and advanced feature modeling strategies.

A foundational effort is the AirBirds dataset by Sun et al. [6], which provides large-scale real-world airport bird image sequences and serves as a benchmark for small-object detection. However, because it was collected from fixed cameras at a single airport, its scene diversity and viewpoint variability remain limited, which inevitably restricts the generalization of models trained on it.

Due to their favorable accuracy–efficiency trade-off, YOLO-based detectors have been widely adopted for airport bird detection [7–9]. Ma et al. [10] enhanced YOLOv8 with RFAConv attention, DyASF-P2 feature fusion, and Inner-ShapeIoU loss, showing improved performance in cluttered scenes. Zhang et al. [11] refined grid partitioning and the detection head, achieving better precision and recall but still suffering from false alarms under strong background interference. Kong et al. [12,13] incorporated CBAM attention and proposed FFC3 and CSPPF modules to balance accuracy and efficiency, though robustness under adverse conditions remains unclear. Sun et al. [14] developed a lightweight YOLOv8 variant by modifying the C2f module, but its cross-scene generalization was not thoroughly evaluated. These studies primarily focus on enhancing feature extraction or detection head design, yet they often address individual components rather than providing a unified solution for small-object detection.

Beyond domain-specific designs, recent work has explored more advanced feature modeling. Xu et al. [15] proposed ESMNet for enhanced feature extraction in complex industrial scenarios, while Li et al. [16] introduced a dynamic multi-scale detection framework to handle significant scale variations. These methods highlight the importance of multi-scale representation and stronger feature modeling capacity, although they are not specifically tailored to airport bird detection.

Despite these advances, several intrinsic limitations remain. First, most methods rely on generic attention mechanisms or shallow feature fusion, which are insufficient for capturing the subtle visual cues of tiny birds (often below 10 pixels) [17]. Second, many multi-scale frameworks retain high-level branches (e.g., P5) that contribute little to small-object detection while increasing computational overhead. Third, widely used IoU-based regression losses struggle to balance localization accuracy and training stability, particularly under low-overlap conditions.

Some studies have incorporated temporal or multimodal information. Sun et al. [18] constructed a large-scale video dataset to enable temporal modeling. Others combined visual data with infrared or radar signals [19,20]. While effective, these approaches often require specialized sensors or additional annotations, limiting their deployment on standard monocular surveillance systems. Segmentation-based methods, such as the CNN–Transformer framework proposed by Gu et al. [21], have also been explored but are generally less suitable for real-time detection due to their high computational cost.

In summary, existing methods have achieved notable progress but still struggle with reliable small-bird detection in complex airport environments. The key challenges are: (i) inadequate fine-grained feature modeling, (ii) insufficient utilization of high-resolution representations, and (iii) unstable localization optimization under low IoU conditions. These challenges directly motivate the design of the proposed RPDL-YOLO framework, which addresses them through backbone enhancement, high-resolution dual-branch detection, and hybrid IoU optimization. A summary of representative methods is provided in Table 1.

**Table 1:** Summary of representative detection methods related to small-object and bird detection.

Method	Core Strategy	Strengths	Limitations
Sun et al. [6]	AirBirds dataset	Real-world benchmark	Limited diversity
Ma et al. [10]	RFAConv + DyASF-P2 + ShapeIoU	Better representation	Noise-sensitive
Zhang et al. [11]	Grid + head redesign	Better P-R balance	High false alarms
Kong et al. [12,13]	CBAM + FFC3 + CSPPF	Balanced accuracy/efficiency	Weak robustness
Sun et al. [14]	Modified C2f	Lightweight	Limited generalization
Xu et al. [15]	ESMNet	Enhanced extraction	Not for bird detection
Li et al. [16]	Dynamic multi-scale	Handles scale variation	Not for bird detection
Gu et al. [21]	CNN-Transformer	Advanced modeling	High cost, not real-time
**RPDL-YOLO**	RepViT + Dual-P2 + Mix_IoU	Strong detection; 93.1 FPS	Slight parameter increase

P-R balance: Precision-Recall balance.

As summarized in Table 1, existing methods mainly focus on improving feature extraction or lightweight design, often addressing these aspects independently. In contrast, the proposed RPDL-YOLO jointly considers fine-grained feature representation, high-resolution detection, and localization stability within a unified framework, leading to more robust performance in complex airport environments.

## Methods

In airport bird monitoring scenarios, bird targets typically exhibit several challenging characteristics, including small object size, rapid motion, irregular flight patterns, and low contrast against complex sky backgrounds. These factors often result in weak feature representation, insufficient localization accuracy, and unstable detection performance in conventional detection models.

To address these challenges, the proposed RPDL-YOLO introduces three targeted improvements. First, the integration of RepViTBlock enhances fine-grained spatial feature representation of small objects. Second, the removal of the redundant P5 detection head and the introduction of a dual-branch high-resolution P2 structure improve the sensitivity to small targets by preserving more spatial information. Third, the Mix_IoU loss function improves localization accuracy and training stability under low-overlap conditions, which are common in small-object detection scenarios.

As illustrated in Fig 1, RPDL-YOLO introduces systematic enhancements to the YOLOv8s framework for small bird detection in airport environments. While retaining the overall Backbone–Neck–Head architecture, the model incorporates targeted improvements at both the structural and training levels to address YOLOv8s’s limitations in fine-grained feature modeling, small-object perception, and localization stability.

**Fig 1:**
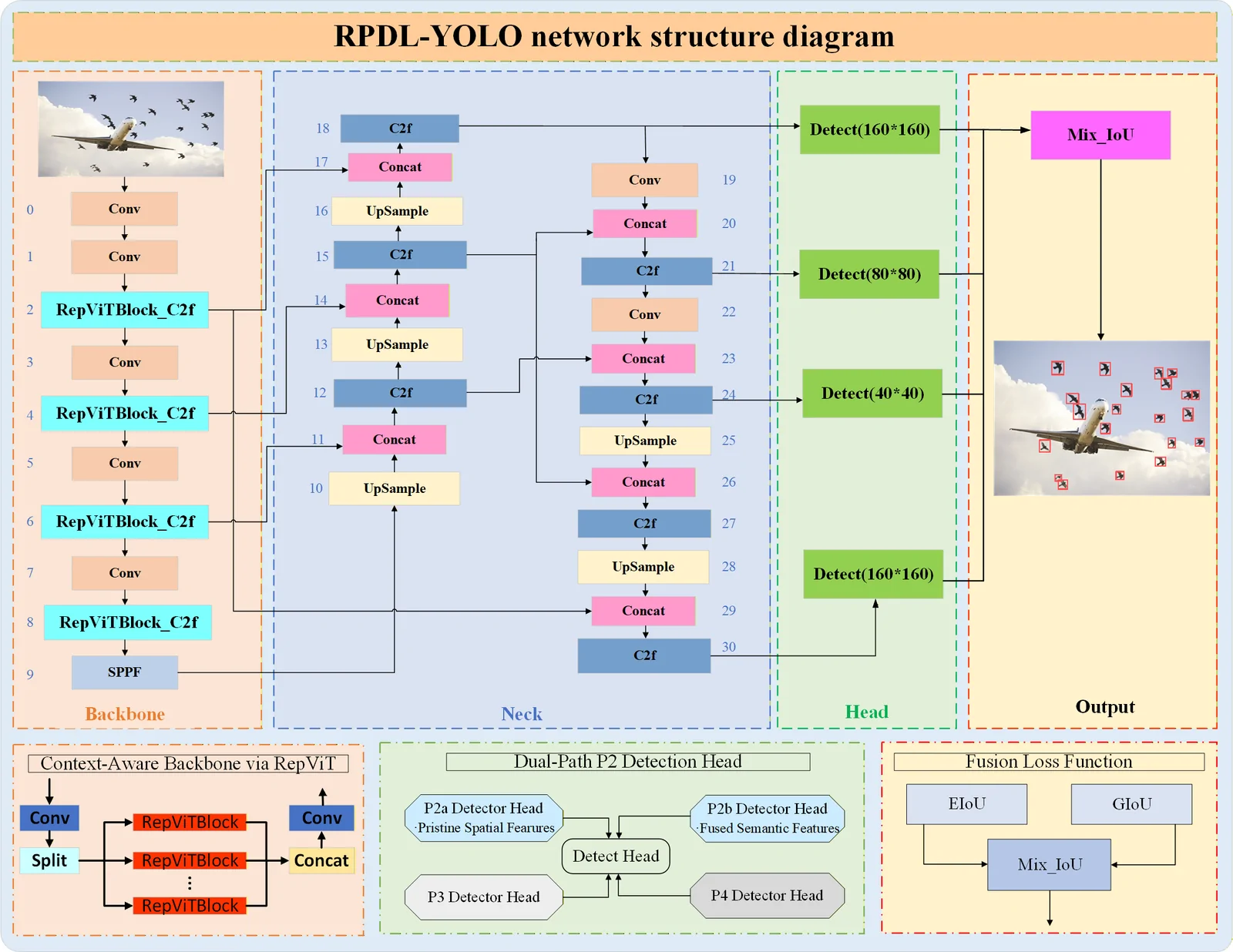
Overview of the proposed RPDL-YOLO detection framework. This figure is generated by the authors.

In the Backbone, the original C2f module is replaced with an enhanced version integrating RepViTBlocks, thereby strengthening the representation of fine-grained spatial features that are essential for recognizing minute bird targets. In the detection head, the redundant P5 detection branch is removed, and a dual-branch structure is introduced at the highest-resolution P2 feature level, enabling the network to more effectively exploit shallow high-resolution features for improved small-object sensitivity. In addition, to enhance localization robustness, the Mix_IoU loss function is adopted to provide more stable supervision for bounding-box regression. These improvements collectively constitute the architectural foundation of RPDL-YOLO, and the subsequent sections describe the design details and functional roles of each component.

### Backbone context enhancement via RepViT integration

#### RepViTBlock: Lightweight token-channel hybrid modeling.

Small bird targets in airport environments often suffer from weak feature representation and insufficient contextual modeling due to their limited pixel information. To address these challenges, the RepViTBlock is introduced to enhance both spatial and channel-wise feature interactions. Conventional convolutional blocks rely primarily on local receptive fields and fixed kernel patterns, which limits their ability to model long-range dependencies and contextual correlations. To alleviate this issue while maintaining computational efficiency, we introduce the RepViTBlock as a lightweight hybrid module that combines convolutional inductive bias with transformer-style feature mixing.

Specifically, each RepViTBlock consists of two sequential components: a *Token Mixer* for spatial interaction and a *Channel Mixer* for inter-channel feature transformation, as shown in Fig 2.

**Fig 2:**
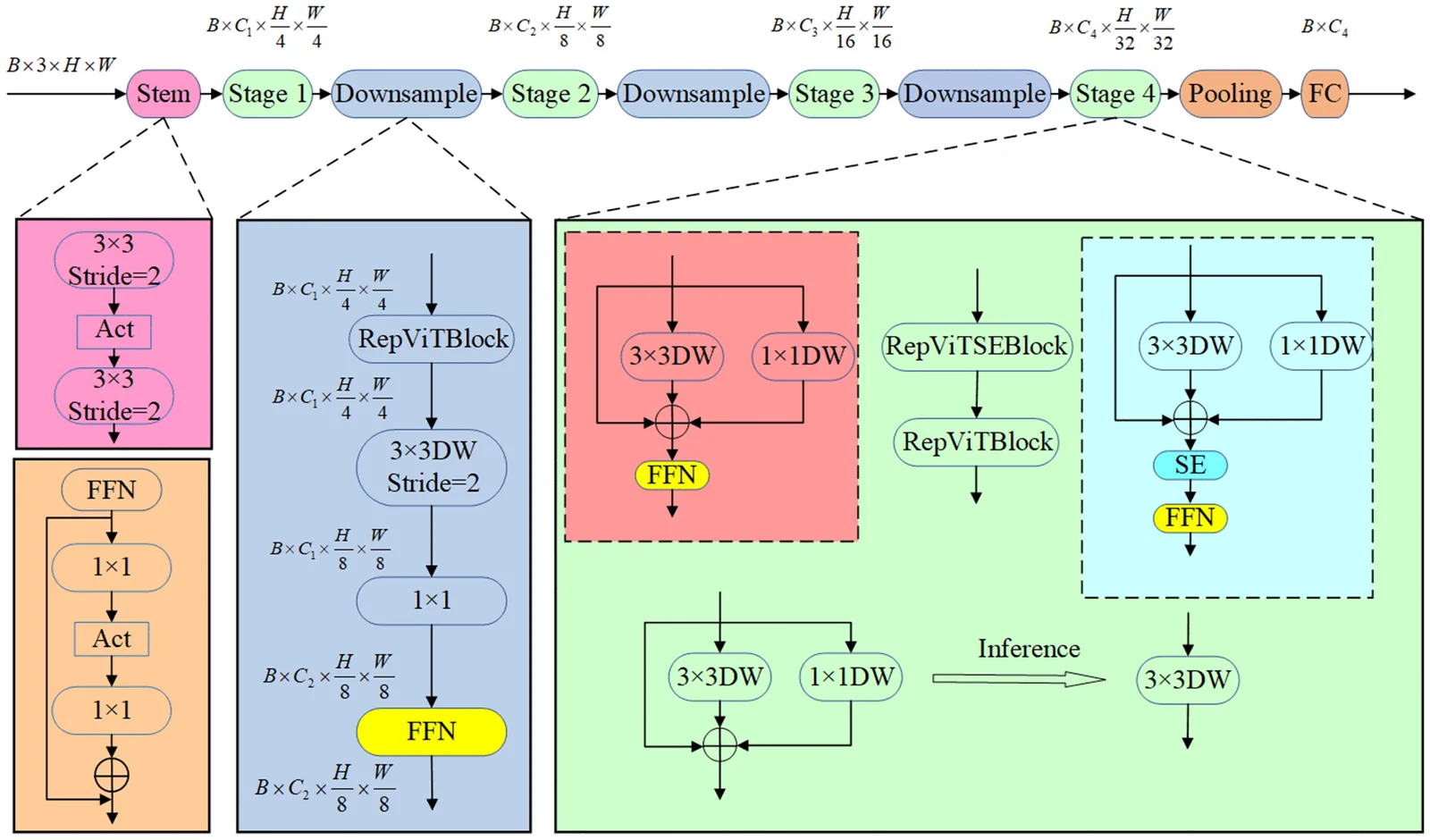
RepViT architecture diagram. Adapted from [22], redrawn by the authors for illustrative purposes.

**(1) Token Mixer**

Spatial token mixing is realized through a RepVGG-style re-parameterizable depthwise convolution structure, expressed as:


$$\begin{array}{c} 𝐓=BN({DWConv}_{3\times 3}(𝐗)+{DWConv}_{1\times 1}(𝐗)+𝐗) \end{array}$$ (1)

where $𝐗\in {\mathbb{R}}^{B\times C\times H\times W}$ is the input feature map, ${DWConv}_{k\times k}(\cdot )$ denotes depthwise convolution with kernel size k×k, BN(·) represents batch normalization, and **T** is the output feature map after spatial token mixing. This design captures fine-grained local spatial patterns while enabling efficient structural re-parameterization during inference.

To further enhance channel-wise response discrimination, a lightweight squeeze-and-excitation (SE) attention mechanism is optionally applied:


$$\begin{array}{c} {𝐓}_{\text{SE}}=SE(𝐓) \end{array}$$ (2)

**(2) Channel Mixer**

The channel mixer adopts a residual feed-forward structure composed of pointwise convolutions:


$$\begin{array}{c} {𝐙}_{1}=\delta ({Conv}_{1\times 1}({𝐓}_{\text{SE}})) \end{array}$$ (3)

$$\begin{array}{c} {𝐙}_{2}={Conv}_{1\times 1}({𝐙}_{1}) \end{array}$$ (4)

where δ(·) denotes the GELU activation function. Equation 3 performs channel-wise feature transformation by first expanding the channel representation through a 1×1 convolution and nonlinear activation, thereby enhancing feature diversity and discrimination across channels. This operation enhances channel-wise feature diversity, thereby improving discriminability for small and visually ambiguous targets.

The final output of the RepViTBlock is obtained via residual aggregation:


$$\begin{array}{c} 𝐘={𝐓}_{\text{SE}}+{𝐙}_{2} \end{array}$$ (5)

Through this design, RepViTBlock achieves efficient spatial-channel feature interaction with minimal computational overhead, making it particularly suitable for high-resolution feature modeling in lightweight detection backbones. This enhanced representation enables the model to better distinguish small bird targets from complex backgrounds, thereby improving detection accuracy and reducing missed detections.

#### RepViTBlock_C2f fusion mechanism.

Conventional CNN backbones in one-stage detectors rapidly downsample input features, discarding high-frequency signals critical for distinguishing sub-10-pixel birds from background textures such as runway markings or grass shadows. While YOLOv8 employs the C2f module to facilitate cross-stage partial feature reuse and efficient gradient propagation, its internal bottleneck units are purely convolutional and lack explicit mechanisms for adaptive spatial–channel interaction. This limitation becomes more pronounced in small object detection scenarios, where subtle texture cues and contextual consistency are critical.

To overcome this limitation, we reformulate the C2f block as a hybrid convolutional–attention unit by embedding lightweight RepViTBlocks—a recent architectural primitive that combines the inductive biases of Vision Transformers with the efficiency of convolutions [22]. Each RepViTBlock integrates depthwise convolutions for local feature extraction and channel-wise attention (via SE modules) to modulate response saliency [23], as widely adopted in recent lightweight detection models [24], thereby enhancing both spatial precision and contextual awareness.

Let the input feature map be


$$\begin{array}{c} 𝐗\in {\mathbb{R}}^{B\times C_{\text{in}}\times H\times W} \end{array}$$ (6)

Following the C2f design, the input is first projected via a 1×1 convolution and then split along the channel dimension:


$$\begin{array}{c} {𝐗}_{0}=[{𝐗}_{a},{𝐗}_{b}] \end{array}$$ (7)

where the split is performed along the channel dimension, with ${𝐗}_{a}$ serving as a shortcut branch that preserves original feature information, and ${𝐗}_{b}$ being progressively refined.

In the proposed design, the transformation branch ${𝐗}_{b}$ is sequentially processed by *n* RepViTBlocks:


$$\begin{array}{c} {𝐘}_{i}={ℛ}_{i}({𝐘}_{i-1}),\;i=1,...,n \end{array}$$ (8)

with ${𝐘}_{0}={𝐗}_{b}$ and ${ℛ}_{i}(\cdot )$ denoting the *i*-th RepViTBlock.

After feature refinement, multi-stage outputs are aggregated with the bypass branch:


$$\begin{array}{c} {𝐗}_{\text{cat}}=Concat({𝐗}_{a},{𝐘}_{1},...,{𝐘}_{n}) \end{array}$$ (9)

followed by a 1×1 convolution to produce the final output:


$$\begin{array}{c} 𝐘={Conv}_{1\times 1}({𝐗}_{\text{cat}}) \end{array}$$ (10)

This fusion strategy offers several advantages:

- **Enhanced Local Representation**: RepViTBlock strengthens fine-grained spatial modeling via re-parameterizable depthwise convolutions.
- **Efficient Channel Interaction**: Lightweight channel mixing improves feature discriminability without introducing heavy self-attention.
- **Stable Gradient Flow**: The original C2f multi-branch aggregation structure is preserved, ensuring training stability.
- **Efficient Deployment**: Structural re-parameterization enables efficient inference, maintaining YOLOv8’s real-time capability.

The proposed RepViTBlock_C2f module effectively balances representational power and computational efficiency, making it particularly suitable for small and morphologically diverse bird detection in complex airport environments. It enables fine-grained spatial detail preservation, which is essential for detecting small and densely distributed targets.

### Dual-path high-fidelity detection at native resolution

Small bird targets often suffer from insufficient spatial detail preservation and limited semantic discrimination in standard detection heads, especially when objects occupy only a few pixels. In the conventional YOLO detection framework, the P3–P5 feature pyramid allocates limited capacity to the highest-resolution level (P2), while the coarsest head (P5) consumes computation without contributing meaningfully to tiny object detection. Moreover, single-branch heads at each scale impose a trade-off between spatial detail and semantic richness, which is particularly detrimental for extremely small targets. To address these limitations, we redesign the detection head to better exploit high-resolution features. While the backbone enhances feature representation, the detection head must effectively utilize high-resolution features for accurate small-object localization.

To address these limitations, we restructure the detection head around the P2 feature map (downsampled by a factor of 4), introducing a novel Dual_P2 architecture composed of two parallel, semantically distinct branches. This design explicitly separates spatial detail preservation from semantic enrichment within the same resolution level, as follows:

- **P2a** operates directly on the unmodified P2 output from the backbone, preserving pristine spatial fidelity to capture fine-grained edges and subtle appearance variations of small birds;
- **P2b** fuses the same P2 map with upsampled semantic context from deeper layers via an auxiliary path in the FPN + PAN neck, enriching it with scene-level understanding to suppress false positives in ambiguous regions (e.g., shadows, debris).

The two branches are implemented with independent parameters to learn complementary representations. This dual-path design enables complementary inference at identical resolution but divergent representational semantics, effectively decoupling spatial detail preservation from semantic representation. As depicted in Fig 3, Dual_P2 focuses on feature refinement at a shared high-resolution level, which is well-suited for small-object detection in airport environments.

**Fig 3:**
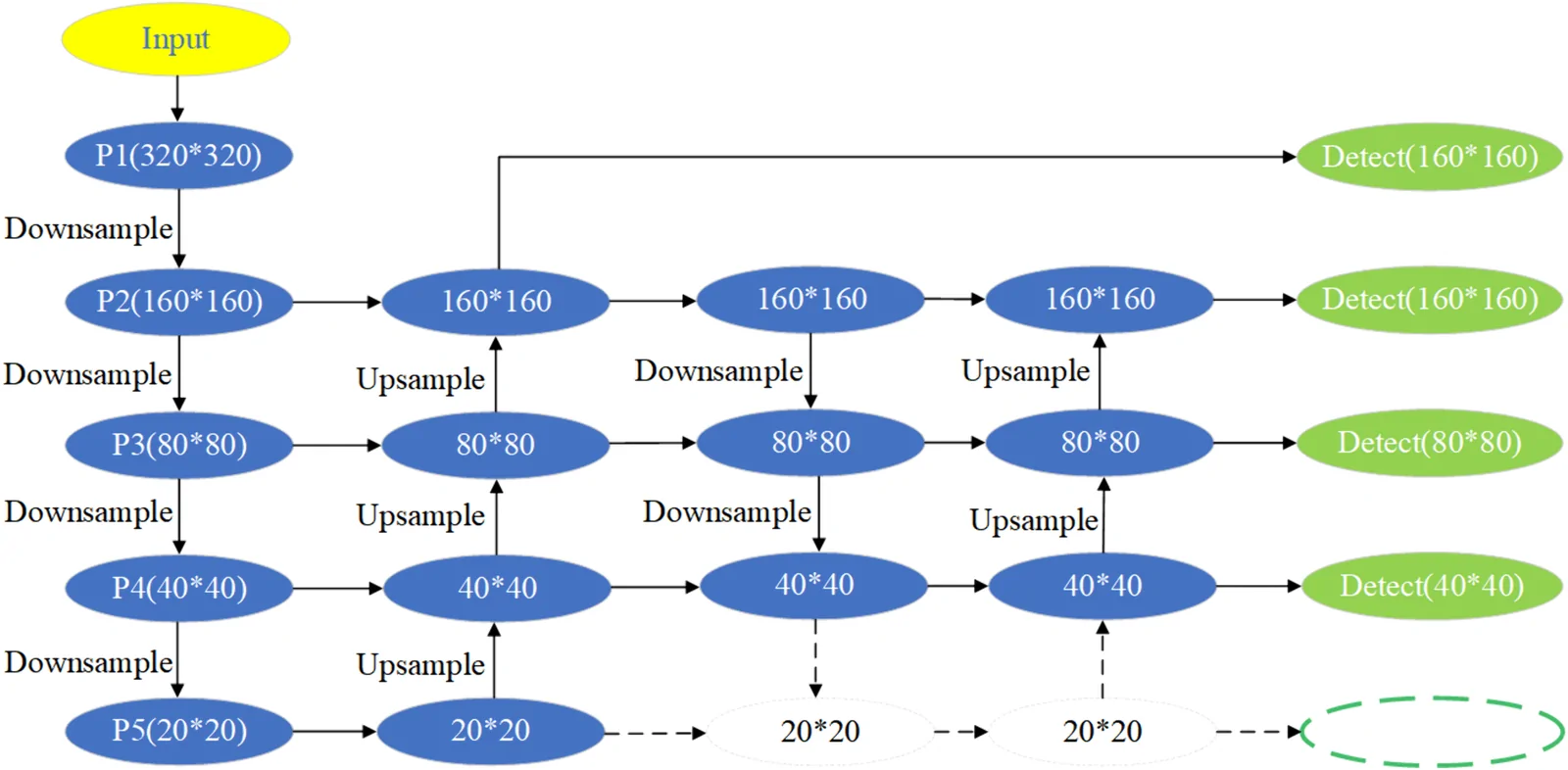
Improved YOLOv8 detector head architecture. This figure is generated by the authors.

The resulting model adopts a four-head design (P2a, P2b, P3, P4), which is tailored to enhance the representation of small and extremely small objects (e.g., birds) while maintaining low computational cost. Overall, this design enhances high-resolution feature utilization and improves the model’s ability to detect small and densely distributed bird targets in complex airport environments.

### Hybrid IoU optimization for robust bounding box regression

While the redesigned detection head improves high-resolution feature utilization, accurate bounding box regression remains challenging for small objects. In small-object detection scenarios, bounding box regression is often unstable due to limited overlap between predicted and ground-truth boxes, especially during early training stages. To address this issue, a more robust loss formulation is required to ensure both stability and accuracy.

EIoU loss enhances bounding box regression by separately optimizing center-point distance, aspect ratio, and scale difference, offering stronger gradient guidance, faster convergence, and higher localization accuracy [25–27]. However, its gradients become unstable when the predicted and ground-truth boxes have minimal or zero overlap, leading to training oscillations. In contrast, GIoU loss introduces a penalty based on the smallest enclosing box, ensuring meaningful gradients even in non-overlapping cases and thus improving training stability. Yet, its coarse modeling of shape and scale limits regression precision.

Recognizing their complementary strengths—the stable gradients of GIoU mitigate the instability of EIoU in low-overlap regions, while the fine-grained optimization of EIoU compensates for the lower accuracy of GIoU—we propose **Mix_IoU**, a weighted combination of EIoU and GIoU. The relative contributions of EIoU and GIoU are controlled by weights α and β, respectively, with α+β=1. The specific weighting scheme is determined through the sensitivity analysis presented in the experimental results. This complementary design maintains stable gradients in low-overlap scenarios while preserving precise localization capability by jointly optimizing EIoU and GIoU. Its formulation is illustrated in Fig 4.

**Fig 4:**
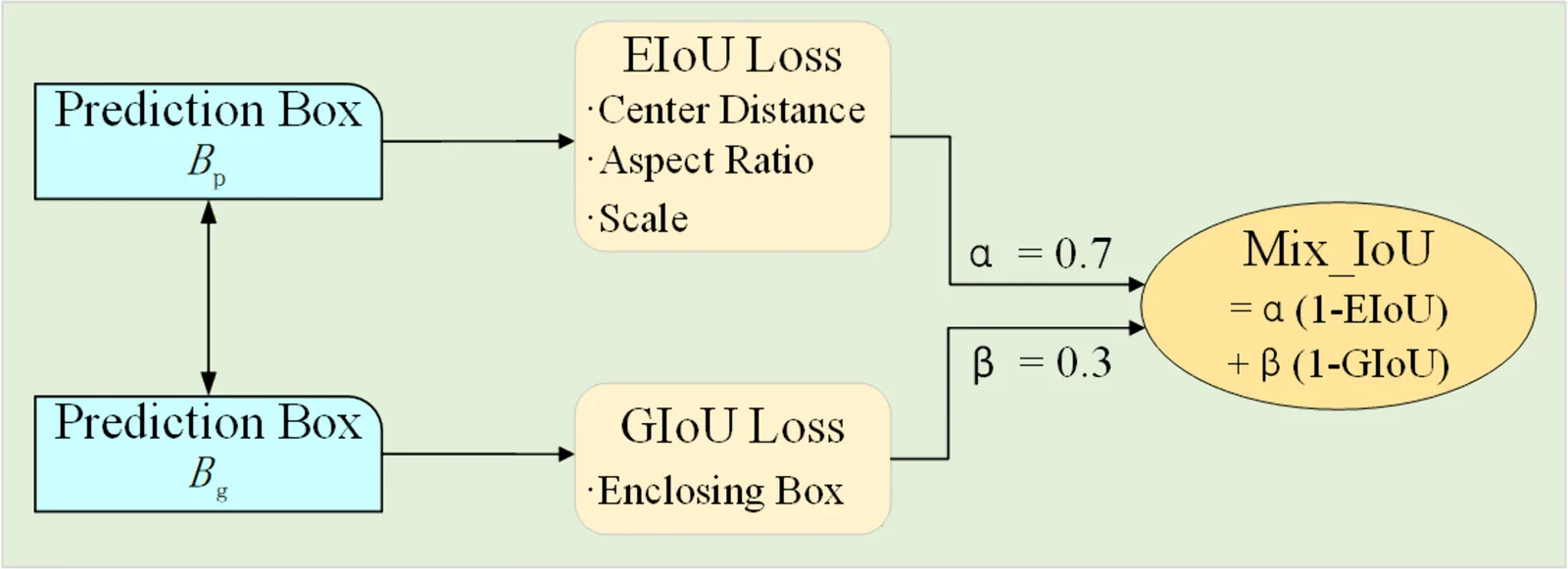
Illustration of the proposed Mix_IoU loss function. This figure is generated by the authors.

**(1) Basic Definitions of EIoU and GIoU**

Let the prediction box be


$$\begin{array}{c} B_{p}=(x_{1}^{p},y_{1}^{p},x_{2}^{p},y_{2}^{p}) \end{array}$$ (11)

The target box is


$$\begin{array}{c} B_{g}=(x_{1}^{g},y_{1}^{g},x_{2}^{g},y_{2}^{g}) \end{array}$$ (12)

The width, height, and center coordinates of the bounding boxes are defined as:


$$\begin{array}{c} w_{p}=x_{2}^{p}-x_{1}^{p},\;h_{p}=y_{2}^{p}-y_{1}^{p} \end{array}$$ (13)

$$\begin{array}{c} w_{g}=x_{2}^{g}-x_{1}^{g},\;h_{g}=y_{2}^{g}-y_{1}^{g} \end{array}$$ (14)

$$\begin{array}{c} b_{p}=\left(\frac{x_{1}^{p}+x_{2}^{p}}{2},\;\frac{y_{1}^{p}+y_{2}^{p}}{2}\right),\;b_{g}=\left(\frac{x_{1}^{g}+x_{2}^{g}}{2},\;\frac{y_{1}^{g}+y_{2}^{g}}{2}\right) \end{array}$$ (15)

The Intersection over Union (IoU) is defined as


$$\begin{array}{c} IoU=\frac{\left|B_{p}\cap B_{g}\right|}{\left|B_{p}\cup B_{g}\right|} \end{array}$$ (16)

GIoU is defined as


$$\begin{array}{c} GIoU=IoU-\frac{\left|B_{c}|-|B_{p}\cup B_{g}\right|}{\left|B_{c}\right|} \end{array}$$ (17)

where $B_{c}$ denotes the smallest enclosing box covering both $B_{p}$ and $B_{g}$. By introducing the enclosing box term, GIoU can still provide effective optimization signals even when the predicted box does not overlap with the ground-truth box.

The EIoU is defined as follows.


$$\begin{array}{c} EIoU=IoU-\frac{\rho^{2}(b_{p},b_{g})}{c^{2}}-\frac{(w_{p}-w_{g}{)}^{2}}{c_{w}^{2}}-\frac{(h_{p}-h_{g}{)}^{2}}{c_{h}^{2}} \end{array}$$ (18)

All geometric terms are normalized by the corresponding dimensions of the enclosing box to ensure scale invariance, where

- $\rho (b_{p},b_{g})$ denotes the Euclidean distance between the centers of the two boxes,
- *c* is the diagonal length of the smallest enclosing box $B_{c}$,
- $c_{w}$ and $c_{h}$ denote the width and height of $B_{c}$, respectively.

This formulation decomposes the localization error into three independent components: the normalized center distance, width difference, and height difference, enabling more precise geometric alignment between the predicted box and the ground-truth box.

**(2) Total Loss**

The Mix_IoU loss computes a weighted combination of EIoU and GIoU for bounding box regression. Given predicted boxes $\left\{B_{p}^{i}\right\}$, ground-truth boxes $\left\{B_{g}^{i}\right\}$, the set of positive samples Ω, weights α and β satisfying α+β=1, and per-sample weights $\left\{w_{i}\right\}$, the bounding box loss ${ℒ}_{\text{bbox}}$ is obtained as follows.

For each positive sample i∈Ω, we first compute the EIoU and GIoU values between the prediction and ground truth, denoted as ${EIoU}_{i}=EIoU(B_{p}^{i},B_{g}^{i})$ and ${GIoU}_{i}=GIoU(B_{p}^{i},B_{g}^{i})$. The per-sample mixed loss is then defined as:


$$\begin{array}{c} {ℒ}_{\text{bbox}}^{i}=\alpha \left(1-{EIoU}_{i}\right)+\beta \left(1-{GIoU}_{i}\right). \end{array}$$ (19)

This per-sample formulation combines the precise localization capability of EIoU with the stable optimization property of GIoU. Finally, the overall loss is normalized by the total sample weights to ensure stability:


$$\begin{array}{c} {ℒ}_{\text{bbox}}=\frac{\sum_{i\in \Omega }w_{i}\;{ℒ}_{\text{bbox}}^{i}}{\sum_{i\in \Omega }w_{i}}. \end{array}$$ (20)

This ${ℒ}_{\text{bbox}}$ is used as the final bounding box regression loss. The proposed Mix_IoU loss improves both training stability and localization precision, making it more suitable for small-object detection under complex conditions.

Based on the above architectural design and module-level formulations, an algorithm-level description is further provided to present the complete workflow of the proposed method. The overall procedure of RPDL-YOLO is summarized in Algorithm 1.

**Algorithm 1 Overall procedure of RPDL-YOLO**

**Require:** Input image **I**

**Ensure:** Detection results **B**

1: Preprocess input image **I**

2: Extract enhanced multi-scale features using RepViT-based backbone

3: Perform feature fusion via FPN + PAN neck

4: Generate detection heads {*P*2*a*, *P*2*b*, *P*3, *P*4}

5: Predict bounding boxes and class probabilities

6: Compute the bounding box regression loss ${ℒ}_{\text{bbox}}$ according to Eq. (20)

7: Output detection results **B**

As shown in Algorithm 1, RPDL-YOLO follows the standard YOLOv8 detection pipeline while introducing targeted improvements in backbone feature extraction, detection head design, and bounding box regression. These modifications are jointly designed to enhance the representation and localization capability for small bird targets in complex airport environments.

### Ethics statement

All data used in this study were collected and processed in accordance with relevant regulations and data usage policies. The public dataset was used in compliance with its official usage terms, and all publicly sourced images were used under their respective licenses. The self-collected data do not contain any personal or sensitive information.

No specific permits were required for this study, as the data collection did not involve any intervention, protected areas, or identifiable human subjects.

## Experimental results and analysis

### Dataset

In this study, target scales are defined based on objects in the image, rather than explicit physical distance. This is because, in practical airport monitoring scenarios, the apparent size of bird targets is influenced by factors such as camera distance, focal length, and viewing angle. Therefore, the scale categories used in this study represent the image-space size of bird targets rather than direct measurements of physical distance. The datasets used in this work include images captured under diverse imaging conditions, which implicitly capture variations in spatial range. Therefore, the proposed scale categorization reflects realistic detection conditions. Nevertheless, we acknowledge that pixel-based scale classification does not explicitly model physical distance. Incorporating camera calibration and distance-aware modeling will be an important direction for future work to further improve real-world applicability.

We evaluated the performance of the proposed RPDL-YOLO model on a bird detection dataset composed of a public benchmark and a small self-collected dataset. The AirBirds dataset is a large-scale dataset specifically designed for real-world flying bird detection to support bird strike prevention [6]. It contains images of diverse bird species captured under various environmental conditions, reflecting realistic airport surveillance scenarios. From the AirBirds dataset, 1,700 images were uniformly sampled for this study. Since most targets in airport scenarios are small objects, we re-annotated a subset of images to improve the labeling accuracy of small and extremely small targets. The re-annotation process was manually performed following a consistent annotation protocol to ensure labeling quality for small targets.

To complement the public dataset, we incorporated additional bird images collected from multiple sources, including high-altitude images obtained from online resources and manually captured images of medium- and large-sized birds. The integrated dataset covers four target scale categories: extremely small, small, medium, and large, with an approximate image-count ratio of 4:3:2:1 across the four categories. This distribution reflects the dominance of small targets in airport bird detection while maintaining multi-scale diversity. To provide a visual understanding of the dataset, representative sample images are shown in Fig 5. The dataset is categorized into four image-space scale levels according to the ratio of the target area to the total image area: extremely small, small, medium, and large. To ensure fairness and reproducibility, all compared methods were trained and evaluated using the same dataset splits and identical training settings. All publicly sourced images are used in accordance with their respective usage licenses.

**Fig 5:**
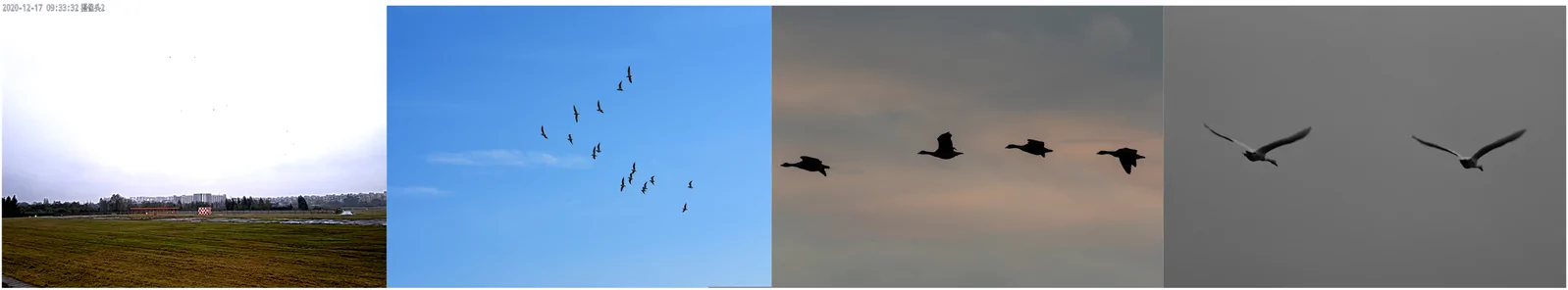
Representative samples of bird targets with different image-space scale categories in the dataset. From left to right: extremely small, small, medium, and large targets. The images are sampled from the dataset used in this study, including the AirBirds dataset, a small portion of self-collected data, and publicly available images from Pixabay (https://pixabay.com), used under the Pixabay License. All images are organized and annotated by the authors.

After dataset integration and augmentation, the final dataset consists of 3,300 annotated images. The dataset was randomly divided into the training set and the test set with a ratio of 8:2, resulting in 2,640 training images and 660 test images, respectively. It should be noted that all experiments in this study were conducted on a workstation platform and are intended solely for algorithm validation. The current work does not involve deployment on UAVs or edge devices; related issues will be further explored in future studies. In addition, all data used in this study comply with the terms and conditions of the original data sources. The public dataset is used in accordance with its official usage policy, while the self-collected data do not contain any personal or sensitive information.

### Evaluation metrics

To comprehensively evaluate detection performance, multiple metrics are adopted, including Precision (P), Recall (R), mAP@50, mAP@50–95, and F1-score. Precision measures the proportion of correct detections among all predictions and is critical for reducing false positives, whereas Recall quantifies the proportion of ground-truth targets that are correctly detected, reflecting the missed detection rate.

The mAP@50 metric evaluates detection accuracy at an IoU threshold of 0.5, whereas mAP@50–95 provides a more comprehensive assessment across multiple IoU thresholds. The F1-score further balances Precision and Recall to indicate overall detection performance. In addition, FPS and GFLOPs are used to evaluate computational efficiency, which is essential for real-time deployment.

### Implementation details

The experiments were conducted on a Windows platform using an NVIDIA GeForce RTX 3060 GPU. The software environment consisted of Windows 11 and PyTorch 2.4.1 with CUDA 11.2. The detailed training parameters are summarized in Table 2.

**Table 2:** Training parameters.

Serial Number	Parameter	Value
1	Number of training epochs	200
2	Image size	640
3	Learning rate	0.0005
4	Patience value	180
5	Optimizer	SGD

Training was conducted with a batch size of 16.

During training, standard data augmentation techniques—including random scaling, horizontal flipping, and mosaic augmentation—were applied to improve robustness to scale variation and background complexity. All models were trained and evaluated using the same data splits to ensure consistency and reproducibility.

### Ablation experiments and analysis

To investigate the contribution of each proposed component, ablation experiments were conducted based on the YOLOv8s baseline. The RepViTBlock_C2f module, Dual_P2 detection head, and Mix_IoU loss were progressively introduced into the network. The quantitative results are summarized in Table 3, including both detection performance and computational efficiency.

**Table 3:** Ablation experiment results.

RepViT	Dual_P2	Mix_IoU	P(%)	R(%)	mAP@50	mAP@50–95	FPS	GFLOPs	F1	Params(M)
			76.6	65.3	75.1	41.5	87.3	21.8	70.5	13.02
✓			78.4	65.9	74.1	40.1	86.7	22.3	71.6	13.78
	✓		77.2	68.8	76.2	43.5	88.2	22.6	72.8	13.95
		✓	79.8	63.8	74.0	41.8	91.8	21.8	70.9	13.02
✓	✓		81.0	68.2	77.4	40.9	89.6	22.9	74.1	14.19
✓		✓	82.8	67.1	79.3	43.9	90.5	22.3	74.3	13.91
	✓	✓	83.2	71.4	78.6	45.9	91.7	22.7	76.7	14.25
✓	✓	✓	84.2	70.7	79.7	46.9	93.1	22.5	76.8	14.50

P%, R%, and mAP@50 denote precision, recall, and mean average precision at IoU threshold 0.5, respectively. FPS indicates frames per second during inference on an NVIDIA GeForce RTX 3060 GPU.

As shown in Table 3, each proposed component contributes differently to performance improvement. The RepViTBlock_C2f enhances fine-grained feature representation, leading to an improvement in precision from 76.6% to 78.4%, primarily by reducing false positives. However, a slight decrease in mAP@50 (from 75.1% to 74.1%) is observed, which may be attributed to stricter feature discrimination that suppresses some borderline detections, thereby affecting overall recall coverage. In contrast, the Dual_P2 structure significantly improves recall and mAP@50, demonstrating its effectiveness in detecting small targets that are easily missed in conventional architectures. The Mix_IoU loss significantly improves precision (to 79.8%) by enhancing localization accuracy. However, it slightly reduces recall when used independently, as stricter localization constraints tend to filter out ambiguous or low-overlap detections, leading to fewer but more reliable predictions. This behavior can be attributed to the improved localization accuracy, which reduces ambiguous predictions, thereby decreasing false positives while filtering out some borderline detections. When combined with other components, it contributes to a more balanced overall performance. In addition, the proposed method achieves improved performance in mAP@50–95, indicating robustness across different IoU thresholds. The model also maintains relatively low computational complexity (22.5 GFLOPs and 14.50M parameters) while achieving 93.1 FPS, demonstrating its suitability for real-time deployment. Overall, the full RPDL-YOLO model achieves a favorable trade-off between detection accuracy and computational efficiency, with RepViTBlock_C2f improving feature representation, Dual_P2 enhancing small-object recall, and Mix_IoU refining localization accuracy.

### Mix_IoU weight sensitivity analysis

To investigate the sensitivity of the proposed Mix_IoU loss to the relative contributions of EIoU and GIoU, six weight combinations were evaluated using the YOLOv8s baseline with Mix_IoU. The EIoU and GIoU weights were set to 0.3/0.7, 0.4/0.6, 0.5/0.5, 0.6/0.4, 0.7/0.3, and 0.8/0.2, respectively. All other model architectures and training settings were kept unchanged, and the results are presented in Table 4.

**Table 4:** Sensitivity analysis of different EIoU and GIoU weights in Mix_IoU.

EIoU Weight	GIoU Weight	P(%)	R(%)	mAP@50	mAP@50–95
0.3	0.7	77.6	62.3	72.8	41.0
0.4	0.6	78.1	62.6	73.1	41.4
0.5	0.5	78.4	62.9	73.2	41.5
0.6	0.4	79.0	63.4	73.7	41.7
**0.7**	**0.3**	**79.8**	**63.8**	**74.0**	**41.8**
0.8	0.2	79.3	63.5	73.6	41.5

α and β denote the weights of EIoU and GIoU, respectively, with α+β=1.

As shown in Table 4, the detection performance changes gradually across the evaluated weight combinations. The 0.7/0.3 configuration achieves the highest Precision, Recall, mAP@50, and mAP@50–95 among the evaluated settings, while further increasing the EIoU weight to 0.8 results in a slight performance decrease. Therefore, α=0.7 and β=0.3 are selected as the default weights for Mix_IoU.

### Scale-wise evaluation

The ablation results in Table 3 demonstrate the overall contributions of the proposed components. To further investigate the detection performance across different target scales and examine the effect of removing the P5 detection head on larger targets, a scale-wise evaluation was conducted on the test set. The bird targets were categorized into four image-space scale levels: extremely small, small, medium, and large, according to the ratio of the target area to the total image area. We report mAP@50 separately for each scale category. The results are presented in Table 5.

**Table 5:** Scale-wise detection performance across different target scales.

Model	Extremely Small	Small	Medium	Large
YOLOv8s	47.8	66.4	80.1	87.2
YOLOv8s w/o P5	48.3	67.6	80.7	86.1
YOLOv8s + Dual_P2	54.7	72.7	82.4	86.2
**RPDL-YOLO**	**59.6**	**75.3**	**84.2**	**88.1**

mAP@50 denotes mean average precision at an IoU threshold of 0.5.

As shown in Table 5, RPDL-YOLO achieves the highest mAP@50 across all four target scale categories. Compared with YOLOv8s, RPDL-YOLO improves mAP@50 by 11.8, 8.9, 4.1, and 0.9 percentage points for extremely small, small, medium, and large targets, respectively. The larger improvements observed for extremely small and small targets indicate that the proposed method provides greater benefits for detecting small-scale bird targets while maintaining comparable performance on larger targets. In particular, removing the P5 detection head causes only a minor decrease of 1.1 percentage points for large targets, from 87.2% to 86.1%, while mAP@50 for extremely small, small, and medium targets slightly increases. Furthermore, the YOLOv8s + Dual_P2 model improves mAP@50 by 6.9 and 6.3 percentage points for extremely small and small targets, respectively, compared with the YOLOv8s baseline. These results demonstrate that the high-resolution Dual_P2 structure contributes more substantially to the detection of small-scale targets, while the removal of the P5 head does not substantially impair detection performance for large image-space targets under the evaluated conditions.

### Comparison with different YOLO variants

#### Quantitative performance comparison.

To further evaluate the effectiveness of the proposed method, RPDL-YOLO is compared with several representative YOLO-based detectors, including YOLOv5s, YOLOv6s, YOLOv8n, YOLOv8s, YOLOv9s, and YOLOv11s. The quantitative comparison results are presented in Table 6.

**Table 6:** Comparative experimental results.

Model	P(%)	R(%)	mAP@50	mAP@50–95	FPS	GFLOPs	F1	Params(M)
YOLOv5s [28]	63.7	50.4	53.0	25.9	83.7	17.1	56.3	7.01
YOLOv6s [29]	67.1	53.5	56.9	27.9	85.0	20.8	59.5	9.11
YOLOv8n [30]	68.2	49.0	52.4	26.2	85.9	10.4	57.0	4.34
YOLOv8s [30]	76.6	65.3	75.1	41.5	87.3	21.8	70.5	13.02
YOLOv9s [31]	74.9	67.6	73.7	41.3	89.1	23.0	71.0	12.80
YOLOv11s [32]	83.0	68.4	80.0	47.1	92.6	21.7	75.0	12.92
**RPDL-YOLO**	**84.2**	**70.7**	79.7	46.9	**93.1**	22.5	**76.8**	14.50

All models were evaluated using the same metrics, dataset, and hardware platform as described for Table 3.

As shown in Table 6, RPDL-YOLO achieves strong overall performance in terms of precision, recall, and mAP@50 while maintaining real-time inference speed. Compared with YOLOv11s, RPDL-YOLO achieves higher precision (84.2% vs. 83.0%) and recall (70.7% vs. 68.4%), while maintaining comparable mAP@50–95 and real-time performance. The slightly lower mAP@50 compared to YOLOv11s (79.7% vs. 80.0%) indicates that the proposed model emphasizes stricter localization, which may reduce marginal detections while improving prediction reliability, particularly for small objects. Overall, RPDL-YOLO achieves a favorable balance between detection accuracy and computational efficiency, demonstrating its suitability for real-time bird detection in airport environments.

Although newer versions such as YOLOv11 incorporate improvements for small-object detection, YOLOv8s is selected as the baseline due to its stable performance and widespread adoption. The proposed improvements focus on enhancing high-resolution feature representation and localization, which are complementary to existing YOLO designs and can be extended to other variants. While this study focuses on comparisons within the YOLO family for controlled evaluation, other frameworks (e.g., transformer-based or two-stage detectors) may provide stronger global modeling capabilities but typically incur higher computational costs, and cross-framework comparisons will be explored in future work.

#### Training curve analysis.

To further analyze the training dynamics of the proposed model, the training performance curves are presented in Fig 6. As observed, the proposed method demonstrates stable convergence behavior with reduced performance fluctuations and improved final accuracy.

**Fig 6:**
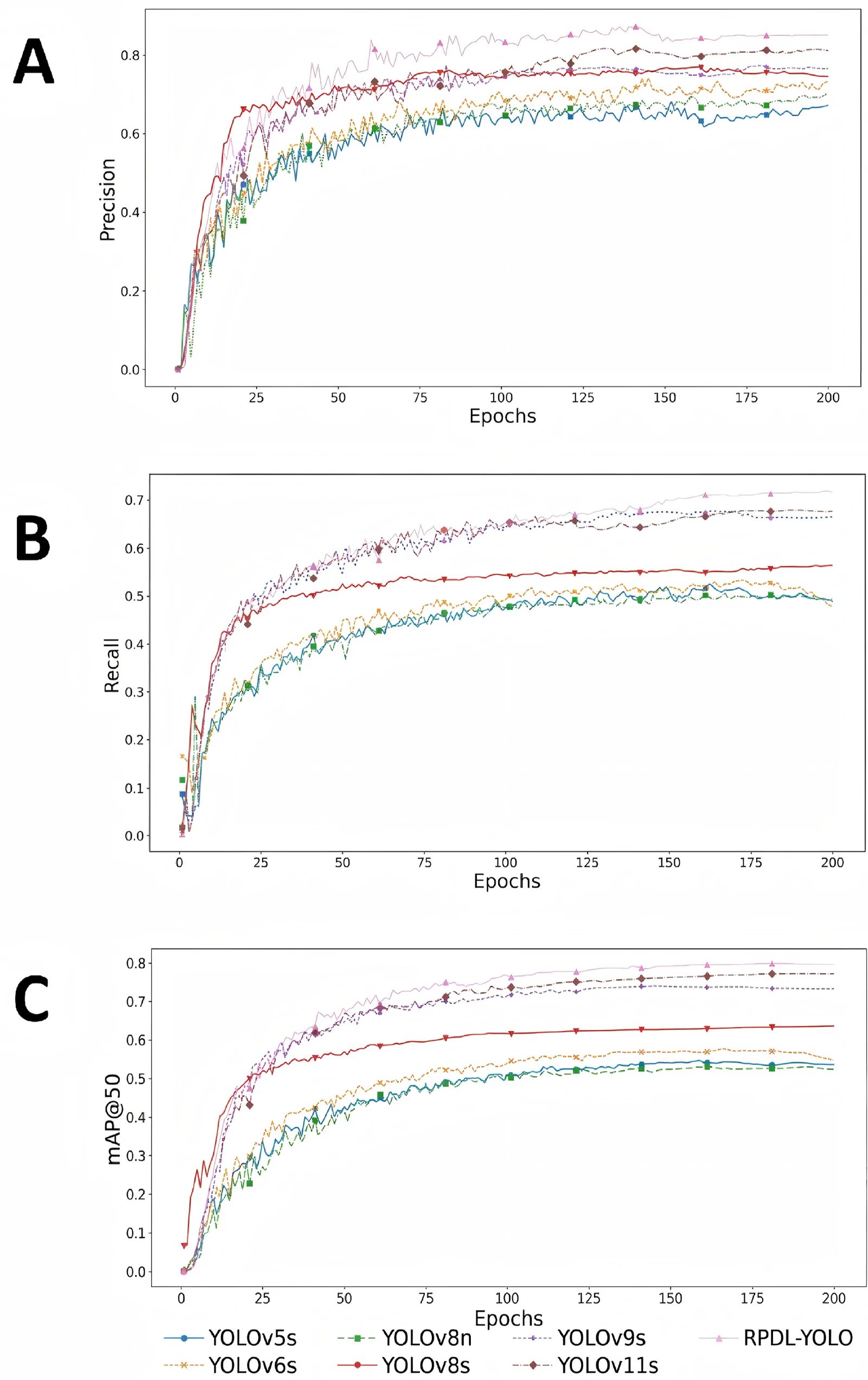
Training performance curves of RPDL-YOLO over 200 epochs. (A) Precision curve, (B) Recall curve, and (C) mAP@50 curve. All curves are generated from the training process of the proposed model by the authors. The results demonstrate the convergence behavior and performance improvement of the model during training.

Compared with baseline models, the improved stability and accuracy can be attributed to enhanced feature representation and more robust optimization introduced by the proposed modules.

### Qualitative results and discussion

#### Visual Comparison Before and After Improvement.

Fig 7 presents qualitative detection results comparing the baseline YOLOv8s and the proposed RPDL-YOLO across multiple representative airport scenarios, including cluttered skylines, distant flocks, and low-contrast backgrounds.

**Fig 7:**
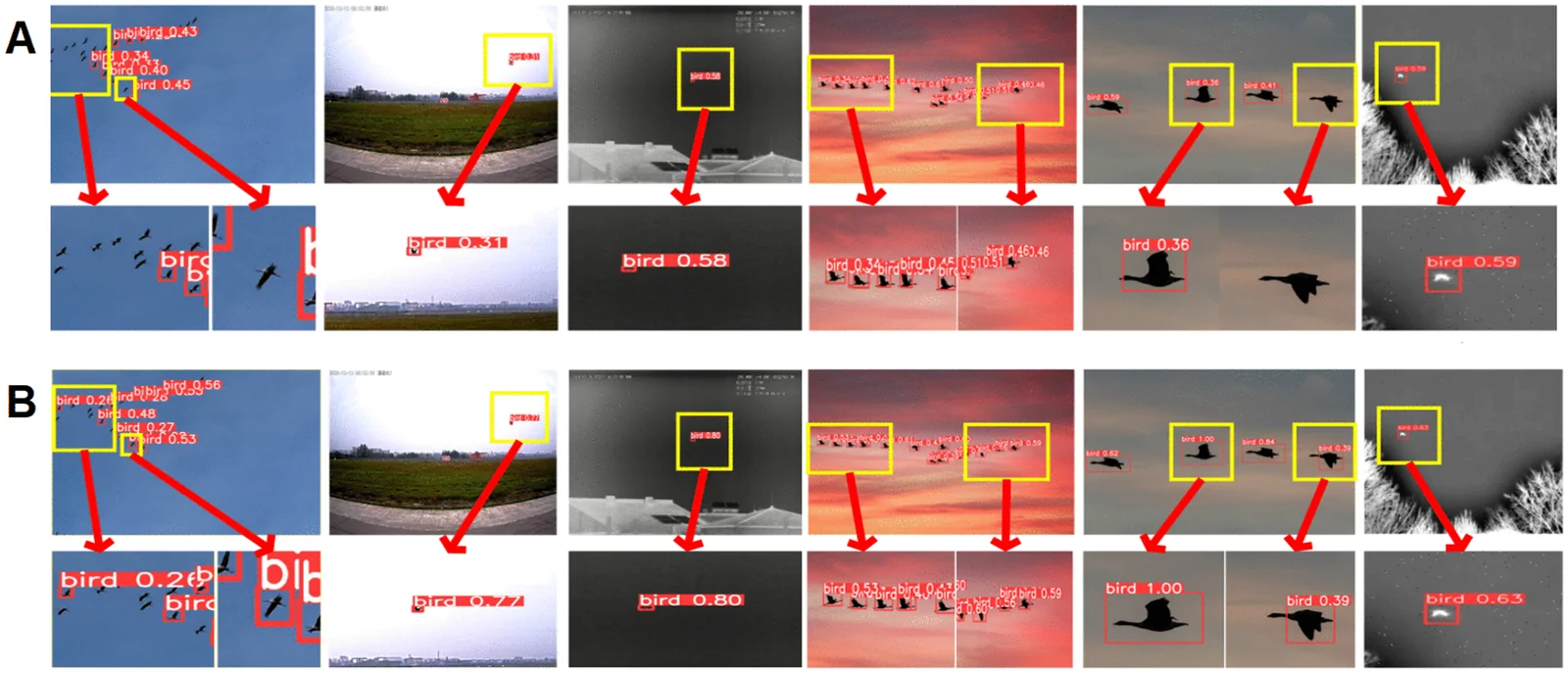
Visual comparison of YOLOv8s and RPDL-YOLO under different scenarios. (A) Detection results of YOLOv8s. (B) Detection results of RPDL-YOLO. The images are sampled from the dataset used in this study, including the AirBirds dataset, a small portion of self-collected data, and publicly available images from Pixabay (https://pixabay.com), used under the Pixabay License. All images are organized and annotated by the authors.

As illustrated, the baseline YOLOv8s exhibits frequent missed detections and low-confidence predictions when targets are extremely small or sparsely distributed. In contrast, RPDL-YOLO consistently detects more targets with higher confidence scores and more compact bounding boxes. This improvement is particularly evident in regions highlighted by the blue and black boxes, where distant or weakly textured birds are partially detected or completely missed by the baseline model.

Overall, these visual comparisons demonstrate that the proposed method substantially enhances detection completeness and confidence for small objects in complex scenes, validating its effectiveness under real-world airport conditions.

#### Detection performance in challenging scenarios.

To further analyze the contribution of individual components, Fig 8 presents qualitative detection results on a challenging scene densely populated with small flying birds. The scene contains numerous targets smaller than 10 pixels against complex sky backgrounds, posing significant challenges for both feature representation and localization accuracy.

**Fig 8:**
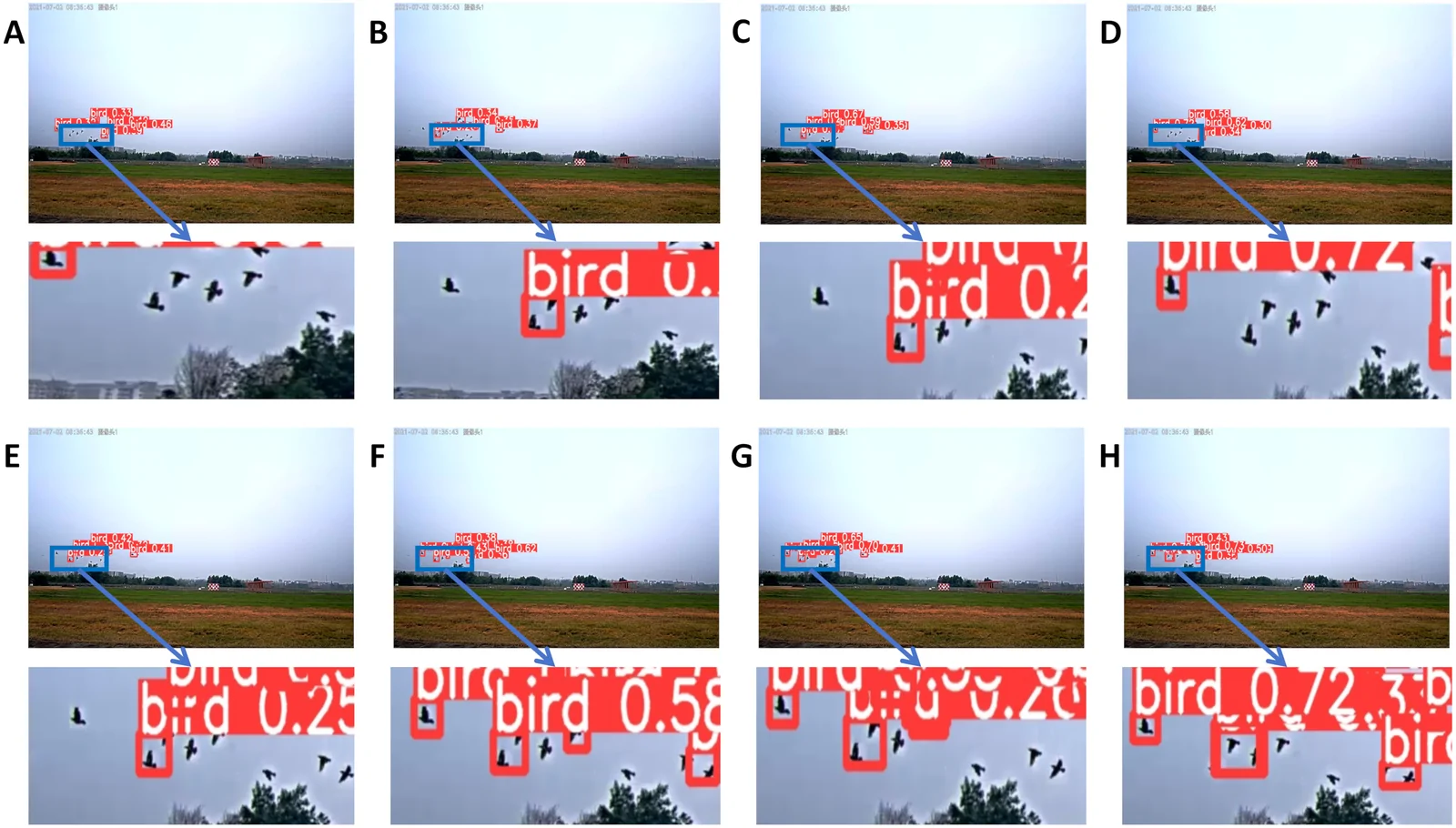
Qualitative comparison of detection results on a challenging sky scene with densely distributed small birds under different ablation settings. (A) YOLOv8s baseline, (B) YOLOv8s + RepViTBlock_C2f, (C) YOLOv8s + Dual_P2, (D) YOLOv8s + Mix_IoU, (E) YOLOv8s + RepViTBlock_C2f + Dual_P2, (F) YOLOv8s + RepViTBlock_C2f + Mix_IoU, (G) YOLOv8s + Dual_P2 + Mix_IoU, and (H) the proposed RPDL-YOLO. The images are sampled from the dataset used in this study, including the AirBirds dataset, a small portion of self-collected data, and publicly available images from Pixabay (https://pixabay.com), used under the Pixabay License. All images are organized and annotated by the authors.

The baseline YOLOv8s suffers from extensive missed detections and unstable confidence scores, especially for distant or partially occluded birds. Introducing the RepViTBlock_C2f module enhances feature discriminability by improving spatial–channel interaction, allowing better separation between birds and background textures. The Dual_P2 head further improves recall by exploiting high-resolution spatial information, enabling the recovery of previously missed small targets. Meanwhile, the Mix_IoU loss stabilizes bounding box regression under low-IoU conditions, leading to more compact localization and increased confidence consistency.

Notably, models combining two components already exhibit visible improvements, while the full RPDL-YOLO model, which integrates all three innovations, achieves the most complete detection coverage with consistently higher confidence scores. This progressive enhancement highlights the complementary nature of the proposed components, which jointly address key challenges in small-object detection, including feature representation, spatial resolution utilization, and localization stability.

## Conclusion

This paper presents RPDL-YOLO, a task-oriented object detection framework for airport bird monitoring, designed to address challenges such as small object size, rapid motion, and complex backgrounds. To overcome these limitations, three complementary improvements are introduced. First, the RepViTBlock_C2f module enhances fine-grained feature representation by integrating lightweight transformer-style modeling into the backbone, improving the discriminability of small and low-contrast targets. Second, a dual-branch high-resolution P2 detection head is designed to better exploit spatial details, improving recall for extremely small objects. Third, the Mix_IoU loss function combines the strengths of EIoU and GIoU, providing more stable optimization and more precise localization under low-overlap conditions. Extensive experiments demonstrate that the proposed method achieves consistent performance improvements over the YOLOv8s baseline, with gains of 7.6% in Precision, 5.4% in Recall, and 4.6% in mAP@50, while maintaining real-time inference speed (93.1 FPS). These results indicate that RPDL-YOLO effectively enhances detection accuracy and robustness, particularly in challenging scenarios involving small, distant, and fast-moving bird targets. From an application perspective, the proposed method provides a practical and effective solution for real-world airport bird monitoring applications, where both high detection accuracy and real-time performance are critical for safety assurance.

Despite these improvements, several limitations remain. First, missed detections may still occur in extremely challenging cases, such as ultra-small targets, low-contrast conditions, and partial occlusions. Second, the current framework operates on single-frame detection and does not explicitly model temporal information, which may limit robustness in dynamic scenes. Furthermore, the model has only been validated on a workstation platform and has not yet been deployed on resource-constrained edge devices, leaving its real-world deployment performance to be further verified. Future work will focus on integrating temporal modeling and tracking to improve detection continuity in dynamic scenarios. In addition, lightweight optimization and hardware-aware design will be investigated to facilitate deployment on UAV and edge devices.

## References

[1] Altringer L, Begier MJ, Washburn JE, Shwiff SA. Estimating the impact of airport wildlife hazards management on realized wildlife strike risk. Sci Rep. 2024;14(1):29018. doi: 10.1038/s41598-024-79946-3 39578506 PMC11584785

[2] Said Hamed Alzadjail N, Balasubaramainan S, Savarimuthu C, Rances EO. A Deep Learning Framework for Real-Time Bird Detection and Its Implications for Reducing Bird Strike Incidents. Sensors (Basel). 2024;24(17):5455. doi: 10.3390/s24175455 39275366 PMC11398100

[3] Mpouziotas D, Karvelis P, Tsoulos I, Stylios C. Automated Wildlife Bird Detection from Drone Footage Using Computer Vision Techniques. Appl Sci. 2023;13(13):7787. doi: 10.3390/app13137787

[4] Liang H, Zhang X, Kong J, Zhao Z, Ma K. SMB-YOLOv5: A lightweight airport flying bird detection algorithm based on deep neural networks. IEEE Access. 2024;12:84878–92. doi: 10.1109/ACCESS.2024.3415385

[5] Zhu J, Ma C, Rong J, Cao Y. Bird and UAVs Recognition Detection and Tracking Based on Improved YOLOv9-DeepSORT. IEEE Access. 2024;12:147942–57. doi: 10.1109/access.2024.3475629

[6] Sun H, Wang Y, Cai X, Wang P, Huang Z, Li D, et al. Airbirds: A Large-scale Challenging Dataset for Bird Strike Prevention in Real-world Airports. In: Proceedings of the Asian Conference on Computer Vision, 2023. 2440–56. 10.48550/arxiv.2304.11662

[7] Wang S, Hao X. YOLO-SK: A lightweight multiscale object detection algorithm. Heliyon. 2024;10(2):e24143. doi: 10.1016/j.heliyon.2024.e24143 38293400 PMC10826665

[8] Tang S, Zhang S, Fang Y. HIC-YOLOv5: Improved YOLOv5 For Small Object Detection. In: 2024 IEEE International Conference on Robotics and Automation (ICRA), 2024. 6614–9. 10.1109/icra57147.2024.10610273

[9] Sun H, Shen Q, Ke H, Duan Z, Tang X. Power Transmission Lines Foreign Object Intrusion Detection Method for Drone Aerial Images Based on Improved YOLOv8 Network. Drones. 2024;8(8):346. doi: 10.3390/drones8080346

[10] Ma J, Guo J, Zheng X, Fang C. An Improved Bird Detection Method Using Surveillance Videos from Poyang Lake Based on YOLOv8. Animals (Basel). 2024;14(23):3353. doi: 10.3390/ani14233353 39682319 PMC11639462

[11] Zhang C, Shi F, Zhang X, Chen S. Airport near-altitude flying birds detection based on information compensation multiscale feature fusion. IEEE Sensors Journal. 2023;23(19):22867–79. doi: 10.1109/JSEN.2023.3304642

[12] Zhang X-W, Kong J-G, Zhang X-W, Zhao Z, Liang X. Real time object detection method for airport flying birds based on improved YOLOv8. Sci Technol Eng. 2024;24(32):13944–52. doi: 10.12404/j.issn.1671-1815.2309930

[13] Zhang X-W, Kong J-G, Zhang X-W, Zhao Z, Liang X. An airport bird detection method based on YOLOv8 network. Telecommun Eng. 2025;65(4):495–502. doi: 10.20079/j.issn.1001-893x.240110002

[14] Sun H, Wang Y, Du J, Wang R. MFE-YOLO: A Multi-feature Fusion Algorithm for Airport Bird Detection. ICCK Trans Intell Syst. 2025;2(2):85–94. doi: 10.62762/TIS.2025.323887

[15] Xu H, Han F, Zhou W, Liu Y, Ding F, Zhu J. ESMNet: An enhanced YOLOv7-based approach to detect surface defects in precision metal workpieces. Measurement. 2024;235:114970. doi: 10.1016/j.measurement.2024.114970

[16] Li Z, Li G, Song X, Wang X. An Efficient and Dynamic Framework for Multi-Scale Target Detection of Underwater Organisms. J Ocean Univ China. 2025;25(1):150–60. doi: 10.1007/s11802-026-6062-9

[17] Shigematsu K. Small bird detection using YOLOv7 with test-time augmentation. arXiv. 2024. doi: arXiv:2401.01018

[18] Sun Z-W, Hua Z-X, Li H-C, Qi Z-P, Li X, Li Y, et al. FBD-SV-2024: Flying Bird Object Detection Dataset in Surveillance Video. Sci Data. 2025;12(1):530. doi: 10.1038/s41597-025-04872-6 40157936 PMC11954963

[19] Ma S, Lu H, Liu J, Zhu Y, Sang P. LAYN: Lightweight Multi-Scale Attention YOLOv8 Network for Small Object Detection. IEEE Access. 2024;12:29294–307. doi: 10.1109/ACCESS.2024.3368848

[20] Zamri FNM, Gunawan TS, Yusoff SH, Alzahrani AA, Bramantoro A, Kartiwi M. Enhanced small drone detection using optimized YOLOv8 with attention mechanisms. IEEE Access. 2024;12:90629–43. doi: 10.1109/ACCESS.2024.3420730

[21] Gu Q, Deng B, He Y, Zhang Y, Cheng L, Wang Y. MarineSeg: A CNN–Transformer Hybrid Architecture with Feature Voting Decoder for Robust Semantic Segmentation in USV-Captured Images. Neurocomputing. 2026;:132597.

[22] Wang A, Chen H, Lin Z, Han J, Ding G. RepViT: Revisiting Mobile CNN From ViT Perspective. In: Proceedings of the IEEE/CVF Conference on Computer Vision and Pattern Recognition (CVPR), 2023. 15909–20. 10.48550/arxiv.2307.09283

[23] Li Z, Xiao L, Shen M, Tang X. A lightweight YOLOv8-based model with Squeeze-and-Excitation Version 2 for crack detection of pipelines. Applied Soft Computing. 2025;177:113260. doi: 10.1016/j.asoc.2025.113260

[24] Hu J, Shen L, Sun G. Squeeze-and-Excitation Networks. In: Proceedings of the IEEE Conference on Computer Vision and Pattern Recognition (CVPR), 2018. 7132–41. 10.1109/CVPR.2018.00745

[25] Tong Z, Chen Y, Xu Z, Yu R. Wise-IoU: Bounding Box Regression Loss with Dynamic Focusing Mechanism. 2023. https://arxiv.org/abs/2301.10051

[26] Zheng Z, Wang P, Liu W, Li J, Ye R, Ren D. Distance-IoU Loss: Faster and Better Learning for Bounding Box Regression. In: Proceedings of the AAAI Conference on Artificial Intelligence, 2020. 12993–3000. 10.1609/aaai.v34i07.6999

[27] Li X, Wang W, Wu L, Chen S, Hu X, Li J, et al. Generalized Focal Loss: Learning Qualified and Distributed Bounding Boxes for Dense Object Detection. In: Advances in Neural Information Processing Systems, 2020. 21002–12. 10.48550/arXiv.2006.04388

[28] Ultralytics. YOLOv5: Real-Time Object Detection. 2020 [cited 2026 April 23]. https://github.com/ultralytics/yolov5

[29] Li C, Li L, Jiang H, Weng K, Geng Y, Li L, et al. YOLOv6: A single-stage object detection framework for industrial applications. arXiv preprint. 2022. doi: arXiv:220902976

[30] Ultralytics. Ultralytics YOLOv8 Object Detection Framework. 2023 [cited 2026 April 23]. https://github.com/ultralytics/ultralytics

[31] Wang CY, Yeh IH, Liao HY. Yolov9: Learning what you want to learn using programmable gradient information. In: European conference on computer vision, Springer. 2024. 1–21.

[32] Ultralytics. YOLO11: Next-Generation Object Detection Models. GitHub repository. 2024 [cited 2026 April 23]. https://github.com/ultralytics/ultralytics

